# Predictors of extra care among magnesium sulphate treated eclamptic patients at Muhimbili National Hospital, Tanzania

**DOI:** 10.1186/1471-2393-11-41

**Published:** 2011-06-03

**Authors:** Projestine S Muganyizi, Mohammed S Shagdara

**Affiliations:** 1 Department of Obstetrics & Gynaecology, Muhimbili University of Health and Allied Sciences (MUHAS), Dar Es Salaam, Tanzania; 2 Department of Obstetrics & Gynaecology, Muhimbili National Hospital, Dar Es Salaam, Tanzania

## Abstract

**Background:**

The inclusion of Magnesium Sulphate (MgSO4) as a gold standard in the treatment of eclampsia has substantially reduced incidences of repeated fits, eclamptic morbidity and deaths. However, despite treatment with MgSO4, a proportion of patients need extra medical/nursing attention and prolonged stay in the intensive care unit (ICU). The literature on the underlying factors for the need of extra care in the MgSO4 era is lacking. This study sought to establish predictors of extra care in ICU among eclamptic patients after treatment with MgSO4 at Muhimbili National Hospital (MNH).

**Methods:**

Data were obtained from hospital records of eclamptic patients who were admitted at MNH and treated with MgSO4 from January 1^st ^to December 31^st^, 2008. Based on set criteria, patients who needed extra care were identified. Analysis was performed using PASW statistics 18 whereby frequencies, cross-tabulations, bivariate and multiple logistic regressions were performed.

**Results:**

A total of 366 eclamptic patients were admitted and treated with MgSO4 at MNH during a 12 month study period in 2008. Most of these (76%) were referred from district hospitals and132 (36%) met the criteria for extra care in ICU. After adjusting for other variables, the risk of extra care in ICU for patients who were admitted with altered consciousness was double (OR = 2.3; 95% CI: 1.3-4.0) that of the ones admitted in alert state. The risk or need of extra care increased by increasing time to delivery and was doubled (OR = 2.0; 95% CI:1.1-3.7) if it was between 12 and 24 hours and tenfold elevated (OR = 10.0; 95% CI:4.3-23.6) if beyond 24 hours as compared to when time to delivery was less than 12 hours.

Abdominal delivery was also independently associated with increased risk compared to vaginal delivery (OR = 2.5; 95%CI: 1.4-4.5). The type of referral and number of fits were associated with extra care in ICU but this association was wholly explained by the clinical status of the patient on admission to MNH and prolonged time lag to delivery.

**Conclusion:**

We concluded that even with MgSO4 used as the gold standard in the treatment of eclampsia, effective pre-referral care and expedited delivery were crucial in minimizing the need for extra care in ICU.

## Background

Eclampsia is a serious obstetric complication which is diagnosed when a woman with signs of pre-eclampsia develops convulsions attributable to the disease [1-3]. Eclampsia is known to complicate 1 in 2000 deliveries in the developed countries and between 1 in 100 and 1 in 1700 in the developing countries thus contributing significantly to the global burden of maternal morbidity and mortality [2-8]. Globally, eclampsia alone accounts for 10% of all maternal deaths [8]. The importance of eclampsia as a cause of severe morbidity is reflected by its inclusion as an indicator in most international measures for severe acute maternal morbidity [9]. At Muhimbili National Hospital (MNH), for many years eclampsia has remained amongst the top 3 causes of maternal mortality and an important cause of maternal morbidity [10,11].

The management of eclampsia has evolved in the past two decades with a wide acceptance and use of Magnesium Sulphate (MgSO4) as the first drug of choice for the control and prevention of eclamptic fits [2,3,12,13]. According to clinical trial reports, MgSO4 is superior to other drugs that were traditionally used to control eclamptic fits such as phenytoin, nimodipine and diazepam [14-16]. In the multinational collaborative eclampsia trial, MgSO4 reduced the risk of recurrent seizures in eclamptic women by 52% when compared to diazepam and by 67% when compared to phenytoin [17]. The publication of these clinical trials significantly increased the acceptance and use of MgSO4 in the treatment of eclampsia [2,3,7,13,18]. In Tanzania, MgSO4 is currently recognized and included in the essential drug list and for over a decade it has completely replaced Diazepam, phenytoin and lytic cocktail for the management of eclampsia at MNH.

The benefits of MgSO4 use in treating eclampsia are not confined to its effectiveness in controlling and preventing eclamptic fits. Studies also show that the use of this drug could further reduce maternal morbidity and mortality. In Nigeria, a study at a tertiary hospital before and after the introduction of MgSO4 reported case specific maternal death reduction from 39.4% to 15% [13]. A similar significant reduction in maternal deaths is reported in Bangladesh [2,3]. Although the majority of eclamptic patients generally recovers quickly and is discharged within a week, about 20-35% remains in hospital for longer periods or suffer added complications that call for extra attention [2,3,19].

The need for extra interventions and prolonged periods of medical/nursing attention among MgSO4 treated eclampsia patients is of great importance particularly in some developing countries like Tanzania where the number of eclampsia cases is relatively high but the resources are limited [2,3,6,8,10,11]. Although few studies have described outcomes of MgSO4 treatment in terms of maternal deaths and associated factors [2,3] the literature on the need and risk factors for extra care among MgSO4 managed eclamptic patients is lacking. This study describes the outcome of MgSO4 treated patients in terms of need for extra care in Intensive Care Unit (ICU) at MNH and factors that predict this need. Our results are expected to set a baseline for future evaluation and improvement of eclampsia management at MNH.

## Methods

### Study settings

This hospital based study consisted of an analysis performed on hospital records of a cohort of mothers who were diagnosed to be eclamptic and who were admitted at MNH and treated with MgSO4 from January 1^st ^to December 31^st^, 2008. MNH is the biggest consultant hospital in the United Republic of Tanzania being situated in Dar es Salaam, the country's largest city. According to the 2002 national population census, the city has a total population of 3.4 million with an annual growth rate of 4.3%. The maternity unit at MNH is affiliated to the department of obstetrics and gynecology and receives referred pregnant and postpartum mothers from Dar es Salaam district hospitals as well as other hospitals from within the city. Occasionally it receives mothers from nearby regions. About 40 women deliver at this unit each day. The MNH also serves as teaching hospital for the Muhimbili University of Health and Allied Sciences (MUHAS) which is public and the largest medical university in the country. This study was ethically approved by the MUHAS ethical committee and permitted by MNH.

### Care of Eclamptic mothers at MNH

Eclampsia patients who are admitted at MNH come with their antenatal cards and a referral note. They are initially registered in the hospital's electronic database which registers their demographic information, referral details and the admitting ward. The patients are then transferred to a special 10 bed ICU where more details are extracted and entered in a ward's register book. Maternal socio-demographic information is detailed in the antenatal card, referral note and patient's case note all of which are retained by the hospital after discharge.

Care in the ICU at MNH is offered by doctors and nurses who are trained in managing eclampsia according to the existing protocol. There is always a rotating specialist in charge of the ward who conducts at least two rounds daily. Any new patient is attended by a resident doctor and the specialist on duty within one hour. Drugs are administered by the nurses who also provide nursing care to the patients. The usual management protocol for eclamptic patients includes intravenous MgSO4 to prevent and control eclamptic fits. A loading dose of 4 g is given slowly intravenously (over 5-10 minutes) followed by a maintenance dose of 1 g hourly for 24 hours (counted from the last fit or delivery). Rapid control of blood pressure is achieved by a slow intravenous Hydrallazine (10 mg) repeated 20-30 minutes to control the diastolic blood pressure between 90-100 mmHg. Long term control of blood pressure is achieved with Methyl dopa. As part of routine care, delivery should be achieved within 12 hours after admission. Induction of labor if necessary is achieved by vaginal Misoprostol or Oxytocin. Any divergence from this protocol is individualized and discussed with a specialist.

Vital signs are checked and recorded on a special sheet half hourly. Number of fits, deep tendon reflexes, urinary protein levels, medication and hourly urine output are also documented. Labor progress and delivery details are recorded on the partogram. Mothers who have delivered and who show good progress in terms of control of fits, improved level of consciousness, improved vital signs including the blood pressure are usually transferred to postnatal ward after completion of a maintenance dose of MgSO4. The decision to transfer a patient to an ordinary postnatal ward is reached based on the specialist's opinion during a ward round and after documentation in the case note. The usual duration of stay in ICU is 48-72 hours regardless of the mode of delivery.

### Data sources

We reviewed hospital admission electronic records, ward admission record book, case notes, partogram records, antenatal cards, and patient care records for all patients admitted to ICU from January 1^st ^to December 31^st^, 2008. A pilot study was conducted at MNH for two months in order to improve our checklist variables and establishing the optimal patient stay in ICU (which was 48-72 hours).

### Data analysis

Data were entered in Epi Info 6 program and cleaned. The software used for analysis was PASW Statistics version 18. In this analysis, parity referred to the number of pregnancies delivered at or after the gestation of 28 weeks excluding the current pregnancy/delivery. The type of referral refers to whether the woman was admitted at MNH before the first fit, or was received from any of the district hospitals, private hospitals or came directly from home. Time taken to delivery was counted from the time the woman was admitted at the referring hospital after fitting or after she developed a fit for the first time while admitted. The criteria for need of extra care in ICU consisted of the presence of any of the following: 1). admission in the ICU beyond 3 days 2). Insertion of nasogastric tube (NGT) 3). Need for resuscitation drugs (such as Calcium gluconate, lasix, adrenaline, hydrocortisone, Oxygen etc) and 4). Need for physiotherapy.

Bivariate logistic regression analysis was conducted to assess the association between variables that characterized each patient and the need for extra care in ICU. The dependent variable was dichotomized into 0 = No need for extra care and 1 = There is need for extra care. All variables with significant p-values (p < 0.05) in the bivariate analysis were included in the multivariate analysis to assess how independently each variable predicted the need for extra care. In the same model, other variables that did not reach a statistical significance level on bivariate analysis but were considered clinically important based on experience were also added. Odds ratios (OR) and 95% confidence intervals (CI) were calculated to estimate the risks for extra care in the study population.

## Results

During the study period, 366 mothers with a diagnosis of eclampsia were admitted to ICU and treated with MgSO4. Among them, there were 18 (4.9%) maternal deaths. Eclamptic mothers were mostly younger than 25 years (68.6%), nulliporous (55.2%), referred from district hospitals (75.7%) and not in labor (73.8%). A total of 75 mothers had incomplete data particularly in terms of gestation age and intrapartum events, hence were excluded in some of the calculations as indicated in Table 1.These 75 mothers, however, were comparable with the rest in terms of age distribution (mean 24 Vs 23.4 years), parity (para < 3 constituted 94% *versus *93%) and need for extra-care in ICU (38% *versus *37%).

**Table 1:** Characteristics of eclamptic patients who were admitted at Muhimbili National Hospital in 2008

Characteristics	Total	Needed Extra care n (%)	Not needed extra care. n (%)
**Age group (years)**			
≤ 19	101	36 (35.6)	65 (64.4)
20-24	150	50 (33.3)	100 (66.7)
25-29	64	27 (42.2)	37 (57.8)
≥30	51	19 37.7)	32 (62.7)
**Parity**			
0	202	69 (34.2)	133 (65.8)
1-2	137	51 (37.,2)	86 (62.8)
≥3	27	12 (44.4)	15 (55.6)
**Marital status**			
Single	31	13 (41.9)	18 (58.1)
Married	161	62 (38.5)	99 (61.5)
Unspecified	174	57 (32.8)	117 (67.2)
**Type of referral**			
Within MNH	32	6 (18.8)	26 (81.2)
Private hospitals	29	17 (58.6)	12 (41.4)
District Hospitals	277	103 (37.2)	174 (62.8)
Home	28	6 (21.4)	22 (78.6)
**Timing of first fit**			
Antepartum	270	104 (38.5)	166 (61.5)
Intrapartum	25	8 (32.0)	17 (68.0)
Postpartum	71	20 (28.2)	51 (71.8)
**Number of fits**			
**1**	94	26 (27.7)	68 (72.3)
2	123	40 (32.5)	83 (67.5)
≥3	149	66 (44.3)	83(55.7)
**State of consciousness**			
Alert	199	56 (28.1)	143 (71.9)
Altered	167	76 (45.5)	91 (54.5)
Gestation age*			
≤32 weeks	63	33 (52.4)	30 (47.6)
33-36	103	39 (37.9)	64 (62.1)
≥37	129	40 (31.0)	89 (69.0)
**Last Hb level (g/dl)**			
> 8.5	313	115 (36.7)	198 (63.3)
≤8.5	53	17 (32.1)	36 (67.9)
**Time taken to delivery†**			
≤12	166	40 (24.1)	126 (75.9)
12:01-24.00	82	35(42.7)	47 (57.3)
≥24.01	43	33 (76.7)	10 (23.3)
**Mode of delivery**			
Vaginal	221	70 (31.7)	151 (68.3)
Abdominal	145	62 (42.8)	83 (57.2)

* Gestation age not known in 71 cases; **† **Labor duration for 75 deliveries not obtained

Overall, 132 (36%) eclamptic mothers needed extra-care in ICU. A typical candidate for extra care in ICU was a parous woman, older than 24 years, referred from a district or private hospital, not alert on admission to MNH and carrying a pre-term pregnancy (Table 1).

The proportion of patients who developed more than two fits before admission to ICU was 12.6% for mothers who fitted for the first time while admitted at MNH, 36.4% for mothers who were admitted directly from home, 41.9% of admissions from district hospitals and 55.1% from private hospitals. Prolonged admission longer than 3 days was the most frequent indicator (33%) of the need for extra care in ICU followed by the use of resuscitation medications (13%) as illustrated in Table 2.

**Table 2:** Incidence of extra care indicators among eclamptic mothers at Muhimbili National Hospital

Indicators Extra care in ICU	Number	Percent (%)
**Days spent in ICU**		
≤ 3	245	66.9
>3	121	33.1
**Needed NGT**		
No	346	94.5
Yes	20	5.5
**Extra-medication**		
No	317	86.6
Yes	49	13.4
**Physiotherapy**		
No	352	96.2
Yes	14	3.8
**Any extra-care needed**		
No	234	63.9
Yes	132	36.1

On bivariate logistic regression analysis, six variables including the type of referral, number of fits, state of consciousness on admission, time to delivery, and mode of delivery and gestation age were significantly associated with need for extra care (Table 3). Variables that were not significantly associated with need for extra care included maternal age (p = 0.67), parity (p = 0.55), marital status (p = 0.43), Hb level (p = 0.51), and timing of first fit in relation to onset of labor (p = 0.25).

**Table 3:** Bivariate and multivariate logistic regression analysis to predict the need for extra care in ICU among eclamptic patients admitted at Muhimbili National Hospital in 2008

Variable	**Needed Extra Care**:		Bivariate Analysis:		Multivariate Analysis:	
	Yes	No	OR	95% CI	OR	95% CI
**Type of referral**						
MNH	6	26	0.9	0.2-3.0	0.3	0.03-3.5
Private hospitals	17	12	5.2	1.6-16.7	3.2	0.7-13.6
District Hospitals	103	174	2.2	0.9-5.5	2.7	0.9-8.7
Home	6	22	1		1	
**Number of fits**						
1	26	68	1		1	
2	40	83	1.3	0.7-2.3	1.3	0.6-2.7
≥3	66	83	2.1	1.2-3.6	1.5	0.8-3.2
**State of consciousness**						
Alert	56	143	1		1	
Altered	76	91	2.1	1.4-3.3	2.3	1.3-4.0
Time taken to delivery*						
≤12	40	126	1		1	
12:01-24.00	35	47	2.3	1.3-4.1	2.0	1.1-3.7
≥24.01	33	10	10.4	4.7-23.0	10.0	4.3-23.6
**Mode of delivery**						
Vaginal	70	151	1		1	
Abdominal	62	83	1.6	1.0-2.5	2.5	1.4-4.5
						
**Gestation Age**						
≤32 weeks	33	30	1		1	
33-36	39	64	0.6	0.3-1.0	0.6	0.3-1.3
≥37	40	89	0.4	0.2-0.8	0.5	0.2-1.0
**Age group (years)**						
≤ 19	36	65	1			
20-24	50	100	0.9	0.5-1.5		
25-29	27	37	1.3	0.7-2.5		
≥30	19	32	1.1	0.5-2.2		
**Parity**						
0	69	133	1			
1-2	51	86	1.1	0.7-1.8		
≥3	12	15	1.5	0.7-3.5		
**Marital status**						
Single	13	18	1			
Married	62	99	1.2	0.5-2.5		
Unspecified	57	117	0.8	0.5-1.2		
**Last Hb level (g/dl)**						
> 8.5	115	198	1			
≤8.5	17	36	0.8	0.4-1-5		
**Timing of first fit**						
Antepartum	104	166	1			
Intrapartum	8	17	0.8	0.3-1.8		
Postpartum	20	51	0.6	0.3-1.1		

*Labor details not available in 75 (20.5%) cases

In a multivariate analysis, all the variables noted to be significantly associated with the need for extra care in ICU on the bivariate analysis were entered in the model. As can be seen in Table 3, after adjusting for other variables, the patient's state of consciousness on admission, time taken to delivery, mode of delivery and gestation age independently predicted the need for extra care in ICU. The importance of the type of referral which was seen on bivariate analysis was contingent to the patient's condition on admission, time taken to delivery and mode of delivery. Likewise the state of consciousness was more important than the absolute number of fits in predicting the need for extra care in ICU.

## Discussion

The general results of this study show that a typical eclamptic patient at MNH was younger than 24 years, nulliporous at the gestational age above 32 weeks and referral from a district hospital. Three quarters of eclamptic mothers were referred after fitting twice or more and nearly two thirds of all eclamptic mothers were admitted with altered consciousness. Late referral of eclamptic patients to MNH can result in increased maternal morbidity and mortality as often observed elsewhere in the developing countries [2,3,7,8,13,19] and previously at the same institution [11]. Moreover, the current study was conducted in the context of low prevalence of provision of parenteral anticonvulsants among health facilities in Tanzania. According to a national survey report [20], only 3% of health centres and 57% of hospitals provide parenteral anticonvulsants for pre-eclampsia/eclampsia treatment. This is in contrary to the Ministry of Health and Social Welfare policy which requires all health centres and hospitals that conduct deliveries to provide parenteral anticonvulsants for the treatment of pre-eclampsia/eclampsia [20].

According to our results, there are two major reasons to doubt the effectiveness of pre-referral management of eclampsia. Firstly, patients who were referred from private and district hospitals suffered disproportionately from repeated fits than those who came directly to MNH from home, implying that measures to prevent repeated fits were not effectively instituted at these hospitals. Secondly, we found a tendency towards increased need for extra care in ICU for patients who arrived at MNH after being cared at other hospitals in contrast to those who came directly from home. For example, an unadjusted risk for extra care in ICU was more than five times if the patient was referred from a private hospital. These discrepancies cannot be explained by the difference in physical accessibility to health facilities since all the district hospitals and most private hospitals are situated within 5-10 kilometres from MNH. Thus patients who came directly to MNH in no way had a better access to health facility than the rest. In the contrary, mothers who attend private hospitals can be assumed to stand a better chance of reporting early to health facilities due to their better economic and social privileges. However, although our arguments are reinforced by the evidence of low prevalence of provision of parenteral anticonvulsants among health facilities in Tanzania [20], we cannot dismiss the possibility that district and private hospital referrals were biased towards complicated cases of eclampsia. Future studies should focus on quality of care of eclamptic patients at these lower level health facilities.

More than a third of eclamptic patients needed extra care in ICU. These mothers because of their severe acute morbidities demanded extra medical and nursing attention and prolonged stay in ICU. Apart from the morbidity it causes, the demand for extra care in ICU can be interpreted into more costs and limit of space in such a busy ICU. It was therefore imperative to understand factors associated with increased demand for extra care in ICU. We found that overall six factors were associated with the demand for extra care including the health facility where the patient came from, number of fits, state of consciousness on admission, duration taken to delivery, mode of delivery and the gestation age. Nevertheless, the importance of the health facility where the patient came from, and the number of fits were contingent on the other four factors on multivariate analysis. This indicates it is the effectiveness of care given to eclamptic patients rather than the place where this care is provided that better predicts the need for extra care in ICU.

Eclampsia require prompt and adequate care if severe complications and mortalities have to be prevented. Provision of MgSO4 immediately after the first fit is the best available means to control and prevent further eclamptic fits and to retain the patient in a good state of consciousness [12]. Studies indicate that provision of even a single loading dose of 14 g MgSO4 is enough to prevent eclamptic fits in the majority [19] but treatment with MgSO4 in late cases may not improve survival [2]. Thus, a single loading dose of MgSO4 can be the minimum recommendation for pre-referral care of eclamptic patients in Tanzania.

As an important component of eclampsia management, it is recommended to terminate pregnancy once the patient is stabilized [7]. According to WHO, it is recommended that termination of pregnancy should be completed within 12 hours once the patient develops eclamptic fits. Some institutions or individuals have become overconfident with MgSO4 and delayed delivery of babies is emerging [2]. In the current study, only 57% of cases delivery was completed in 12 hours after admission. In 15% of the cases, delivery was completed longer than 24 hours. Nevertheless, our results indicate progressive increase in the risk for extra care in ICU for mothers who were undelivered in less than 12 hours with the most drastic risk (10 times) observed among mothers who were undelivered for more than 24 hours. These results underscore the need for expedite delivery of eclamptic mothers at MNH.

The eclampsia case fatality rate of 4.9% in the current study was close to the previously reported rate of 5.0%-7.7% at the same hospital [10,11] indicating that there has not been a significant improvement in the reduction of maternal mortality due to eclampsia for the past 10 years. Although this case specific fatality seems much higher than what is reported in the developed countries such as the 0.4% in Canada[21], it is lower than what is often reported in some other developing countries including the 29.5% in India[22], 7.8-39.4% in Nigeria[13,23-25] and 10.7% in Benin[26]. Moreover, the 4.9% mortality due to eclampsia is within the internationally acceptable eclampsia specific maternal mortality of less than 5% [7,27]. Nevertheless, the variations in maternal mortality rates due to eclampsia indicate that, there is room for further reduction of eclampsia related maternal mortality at MNH.

Like other retrospective studies, the current study suffered incompleteness of data in the case records and in other documents that were used. Thus, some of the important information such as socioeconomic background, treatment details, timing of fits at home, and timing of attendance by the specialists and by others, details for postpartum eclampsia cases and others were lacking. Another limitation was related to the sample size. Since some of the indicators of the need for extra care in ICU were not very prevalent, the adverse events were few leading to the wide 95% confidence intervals in some regression analyses. In order to overcome these problems, a multicenter prospective study is suggested that will provide a much more detailed data for Tanzania.

## Conclusions

In conclusion, the proportion of eclamptic mothers who need extra care in ICU at MNH is high despite of the availability of MgSO4 which is currently the gold standard in controlling eclamptic fits. Ineffective pre-referral management of eclampsia and the failure to expedite delivery are the key underlying factors. Future interventions should target community education and improvement of case management at all the referral facilities including MNH.

## Competing interests

The authors declare that they have no competing interests.

## Authors' contributions

PSM participated in conception, study designing, supervision of data collection, analysis, interpretation of data, drafting the manuscript and final approval of the manuscript for publication.

MSS participated in conception, study designing, data collection, analysis, interpretation of data, and final approval of the manuscript for publication.

## Pre-publication history

The pre-publication history for this paper can be accessed here:

http://www.biomedcentral.com/1471-2393/11/41/prepub

## References

[B1] Obed SA Wilson JB Sakay A Determinants of maternal mortality in eclampsia at Korle Bu Teaching Hospital Accra, Ghana Ghana Med J 1999 33 86 89

[B2] Begum MR Begum A Quadir E Akhter S Shamsuddin L Eclampsia: Still a problem in Bangladesh MedGenMed 2004 6 4 52 15775879 PMC1480554

[B3] Begum R Begum A Bullough CH Johanson RB Reducing maternal mortality from eclampsia, using magnesium sulphate Eur J Obstet Gynecol Reprod Biol 2000 92 2 223 224 10.1016/S0301-2115(99)00274-2 10996685

[B4] Douglas KA Redman CWG Eclampsia in the United Kingdom BMJ 1994 309 1395 1400 7819845 10.1136/bmj.309.6966.1395 PMC2541348

[B5] World Health Organization International Collaborative Study of Hypertensive disorders of Pregnancy. Geographic variation in the incidence of hypertension in pregnancy Am J Obstet Gynecol 1988 158 80 83 2962500

[B6] Bergstrom S Povey G Songane F Ching C Seasonal incidence of eclampsia and its relationship to meteorological data in Mozambique J Perinat Med 1992 20 153 158 1501059 10.1515/jpme.1992.20.2.153

[B7] Choudhary P Eclampsia: a hospital based retrospective study Kathmandu University Medical Journal 2003 1 4 237 241 16388262

[B8] Duley L Maternal mortality associated with hypertensive disorders of pregnancy in Africa, Asia, Latin America and the Caribbean Br J Obstet Gynaecol 1992 99 547 10.1111/j.1471-0528.1992.tb13818.x 1525093

[B9] Say L Pattinson RC Gülmezoglu AM WHO systematic review of maternal morbidity and mortality: the prevalence of severe acute maternal morbidity (near miss) Reproductive Health 2004 1 3 10.1186/1742-4755-1-3 15357863 PMC516581

[B10] Kidanto H Mogren I Massawe S Lindmark G Nystrom L Criteria-based audit on management of eclampsia patients at a tertiary hospital in Dar es Salaam, Tanzania BMC Pregnancy Childbirth 2009 9 13 10.1186/1471-2393-9-13 19323846 PMC2670267

[B11] Urassa D Carlstedt A Nyström L Massawe S Lindmark G Eclampsia in Dar es Salaam, Tanzania: incidence, outcome, and the role of antenatal care Acta Obstet Gynecol Scand 2006 85 5 571 578 10.1080/00016340600604880 16752236

[B12] Euser A Cipolla M Magnesium sulfate treatment for the prevention of eclampsia: A brief review Stroke 2009 40 4 1169 1175 10.1161/STROKEAHA.108.527788 19211496 PMC2663594

[B13] Tukur J Muhammad Z Management of eclampsia at AKTH: before and after magnesium sulphate Niger J Med 2010 19 1 104 107 20232764 10.4314/njm.v19i1.52492

[B14] Belfort M Anthony J Saade G Allen J The Nimodipine Study Group. A comparison of magnesium sulfate and nimodipine for the prevention of eclampsia N Engl J Med 2003 348 304 311 10.1056/NEJMoa021180 12540643

[B15] Duley L Henderson-Smart D Magnesium sulphate versus phenytoin for eclampsia Cochrane Database Syst Rev 2003 4 10.1002/14651858.CD000128 14583911

[B16] Lucas M Leveno K Cunningham F A comparison of magnesium sulfate with phenytoin for the prevention of eclampsia N Engl J Med 1995 333 201 205 10.1056/NEJM199507273330401 7791836

[B17] The Eclampsia Trial Collaborative Group Which anticonvulsant for women with eclampsia? Evidence from the collaborative eclampsia trial Lancet 1995 345 1455 1463 7769899

[B18] Gülmezoglu A Duley L Use of anticonvulsants in eclampsia and pre-eclampsia: Survey of obstetricians in the United Kingdom and Republic of Ireland BMJ 1998 316 975 976 9550956 10.1136/bmj.316.7136.975 PMC28500

[B19] Ekele B Muhammed D Bello L Namadina I Magnesium sulphate therapy in eclampsia: the Sokoto (ultra short) regimen BMC Res Notes 2009 19 2 165 10.1186/1756-0500-2-165 PMC2732634 19691837

[B20] National Bureau of Statistics (NBS) (Tanzania) and Macro International Inc Tanzania Service Provision Assessment Survey 2006 2007 Dar es Salaam, Tanzania: National Bureau of Statistics and Macro International Inc

[B21] Wen S Huang L Liston R Severe maternal morbidity in Canada, 1991-2001 CMAJ 2005 173 7 10.1503/cmaj.045156 PMC1216316 16186582

[B22] Swain S Ojha K Prakash A Bhatia B Maternal and perinatal mortality due to eclampsia Indian Pediatr 1993 30 6 771 773 8132257

[B23] Igberase G Ebeigbe P Eclampsia: ten-years of experience in a rural tertiary hospital in the Niger delta, Nigeria Journal of obstetrics and gynecology: the journal of the Institute of Obstetrics and Gynecology 2006 26 5 414 417 10.1080/01443610600720113 16846866

[B24] Kullima A Kawuwa M Audu B Usman H Geidam A A 5-year review of maternal mortality associated with eclampsia in a tertiary institution in northern Nigeria Annals of African Medicine 2009 8 2 81 84 10.4103/1596-3519.56233 19805936

[B25] Ozumbia B Ibe A Eclampsia in Enugu, eastern Nigeria Acta Obstet Gynecol Scand 1993 72 3 189 192 10.3109/00016349309013370 8385854

[B26] Onuh S Aisien A Maternal and fetal outcome in eclamptic patients in Benin City, Nigeria Jobstet Gynaecol 2004 24 7 765 768 10.1080/01443610400009451 15763783

[B27] Raksha A Gangul RP Swain S Oumachiqul Raja Ram P Determinants of maternal mortality in eclampsia in India Australian NZJ Obstet Gynaecol 1994 34 537 539 10.1111/j.1479-828X.1994.tb01104.x

